# Association of Glycosylated Haemoglobin (HbA1c) Level With Left Ventricular Diastolic Dysfunction in Patients With Type 2 Diabetes

**DOI:** 10.7759/cureus.31626

**Published:** 2022-11-17

**Authors:** Rishi T Guria, Manoj K Prasad, Brajesh Mishra, Sujeet Marandi, Amit Kumar, Ajit Dungdung

**Affiliations:** 1 Internal Medicine, Rajendra Institute of Medical Sciences, Ranchi, IND; 2 Medicine, Rajendra Institute of Medical Sciences, Ranchi, IND; 3 Pulmonary Medicine, Rajendra Institute of Medical Sciences, Ranchi, IND; 4 Laboratory Medicine, Rajendra Institute of Medical Sciences, Ranchi, IND

**Keywords:** echocardiography, fasting blood sugar, type 2 diabetes mellitus (type 2 dm), left ventricular diastolic dysfunction (lvdd), glycosylated haemoglobin (hba1c)

## Abstract

Background

Some literature has shown a high prevalence of pre-clinical diastolic dysfunction in subjects with type 2 diabetes mellitus. The current study was carried out to determine the association of glycosylated hemoglobin (HbA1c) levels with left ventricular diastolic dysfunction (LVDD) in patients with type 2 diabetes.

Methods

An observational cross-sectional study was conducted in a tertiary health care center in Jharkhand. A total of 100 subjects diagnosed with type 2 diabetes mellitus who gave informed consent and fulfilled the inclusion and exclusion criteria were studied in our center from April 2019 to September 2020. Logistic regression was carried out to determine the association of potential variables with outcomes. Multivariable logistic regression analysis was conducted to determine the independent effects of variables for LVDD prediction.

Results

The mean HbA1c of the population with LVDD was found to be higher (11.07 ± 3.66%) as compared to the population with normal LVDD (9.11 ± 2.95%), which was found statistically significant (probability value (P) =0.004). This signifies that a higher level of HbA1c in a patient with diabetes will have a higher incidence of LVDD. On applying multivariate analysis to determine the independent effect of variables for LVDD, HbA1C was found to be significant with an odds ratio (OR) of 1.26, 95% CI 1.08-1.48. The duration of diabetes was also found to be significant with OR 1.48 and CI 95 % (1.20-1.82) P <0.001. On plotting the receiver operating characteristic curve (ROC), the area under the ROC curve to predict the left ventricular function with the model was 0.8137.

Conclusions

Patients who have higher HbA1C are linked to a higher risk of left ventricular diastolic dysfunction in patients with type 2 diabetes. The combination of diabetes and left ventricular dysfunction can lead to increase morbidity and mortality in those patients in whom it is not identified timely and appropriate measures are not taken. Our work emphasizes the requirement of screening intermittently symptomless diabetic patients for diastolic dysfunction through a Doppler echocardiography so that timely action can be taken.

## Introduction

Diabetes mellitus is a group of diseases where there is a chronic elevation of blood sugar. It is usually due to impaired insulin secretion and action. India is considered to be the world's capital of diabetes. The diabetic population in the country is going to hit the disquieting mark of 80 million by 2030 [1].

The widespread presence of diabetes mellitus is escalating throughout the world. The prevalence of type 2 diabetes mellitus is rising more rapidly, due to escalating obesity and decreased physical activity. Few studies have revealed a high prevalence of pre-symptomatic diastolic dysfunction in subjects of diabetes mellitus [2].

Diabetes is one of the prime risk factors for diastolic heart failure. In patients with diastolic heart failure, the mortality rate ranges from 5-8% annually, whereas in systolic heart failure, it ranges from 10-15% [3]. Studies suggest that in diabetic patients, myocardial damage affects diastolic function earlier than systolic function. The impaired filling of the heart during diastole is described as diastolic dysfunction. An initial pressure gradient difference between the left atrium (LA) and the left ventricle (LV) causes the filling of blood in the left ventricle. Especially ventricles of young healthy individuals also create suction in the very early phase of filling due to a rapid expansion of the left ventricle. In between passive and active filling of the left ventricle, there is a period where only a little filling occurs, the so-called diastasis. Therefore, diastolic dysfunction is understood as impaired left ventricular relaxation with increased stiffness of the left ventricle and elevated filling pressures. The etiology of left ventricular dysfunction in diabetic patients is not clearly known.

There are suggested underlying mechanisms, such as microvascular disorder, autonomic breakdown, and metabolic and interstitial fibrosis, for diabetic cardiomyopathy although it is an independent cardiovascular disease [4]. The first stage of diabetic cardiomyopathy is left ventricular diastolic dysfunction. It precedes changes in systolic function, emphasizing the early assessment of ventricular function in people with diabetes [5]. Even in the absence of diabetic complications of the cardiovascular system, there can be diastolic abnormalities in diabetic patients [6].

Till now, no studies have been done in Jharkhand state to see the prevalence of LVDD in diabetics. The association of LVDD with glycemic control is even now a concern. So, this cross-sectional study was carried out to determine the prevalence of asymptomatic LVDD in normotensive subjects of type 2 diabetes mellitus and its relation to HbA1c.

## Materials and methods

Study design

The present work was approved by the Institutional Ethical Committee, Rajendra Institute of Medical Sciences, Ranchi, Jharkhand, India (no 11/IRC/RIMS, dated 16.02.2019). The patients or their relatives were counseled and written informed consent was taken from the subject or their relatives with their signature. All the participants had the option to withdraw from the study at any point without giving any reason or prior notice.

This cross-sectional observational study was conducted from April 2019 to September 2020, on patients with type 2 diabetes who met the inclusion and exclusion criteria. Inclusion criteria included patients aged greater than 18 years and less than 65 years, with a blood pressure of <130/80 mmHg measured three times, normal electrocardiogram (ECG), and no cardiovascular symptoms. Cases were selected as per the diagnostic criteria laid down by the American Diabetes Association. Fasting blood glucose (FBS) of 126 mg/dl or higher or a 2-hr postprandial blood glucose (PPBS) level of 200 mg/dl or higher during a 75-gm oral glucose tolerance test or random plasma glucose of 200 mg/dl or higher in a patient with symptoms of hyperglycemia or HbA1c 6.5% or higher.

Exclusion criteria consist of systemic arterial hypertension, congestive heart failure, ischemic heart disease, valvular heart disease, cardiomyopathy, connective tissue disease, chronic kidney disease, chronic liver disease, and thyroid dysfunction.

Patient data were collected and recorded as per a prepared proforma for each patient. Each subject was given a unique serial number and enrolled in the study as a case. Clinical history-taking, thorough clinical assessment, and an investigation of FBS, PPBS, and HbA1c were done for all the participating subjects.

The HbA1c test was done by using the ion-exchange high-performance liquid chromatography method (HPLC). The tests done to assess cardiac dysfunctions were electrocardiogram (ECG) and 2D echocardiography. In this study, a 2D echocardiography machine was used with an adult cardiac probe: Electronics Phased Array probe (Starmans Electronics S.r.o., Prague, Czechia) with 512 electronic independent channels. Imaging modes were dual 2D real-time, 2D and M mode, color Doppler, pulsed wave Doppler, and continuous wave Doppler. As per the recommendations of the American Society of Echocardiography, all the measurements and recordings were taken by the same observer. Patients were classified into two groups, those having LVDD and those not having LVDD according to guidelines for the evaluation of left ventricular diastolic function by echocardiography [7].

The sample size was calculated using the formula n=4pq/d2 n=sample size p=prevalence of LVDD in type 2 diabetes mellitus (found to be a pooled prevalence of 44% by earlier studies [8-24], q=100-p d=.10 (considering a precision of 10%), so n=4x44x56/10x10 =98, thus rounding the final sample size as 100.

Statistical analysis

A descriptive statistical analysis was carried out and results on continuous measurements were categorized across various value ranges and described in number (%). A logistic regression analysis was done to select the potential variables suitable to determine the association in the multivariable analysis. To determine the independent relationship of potential variables for the risk of LVDD multivariable logistic regression analysis was carried out. The predictive accuracy of the multivariable model was assessed by the logistic receiver operating curve. All statistical analyses were done by statistical software STATA version 13 (College Station, TX: StataCorp LP).

## Results

A hundred subjects with type 2 diabetes who fulfilled the inclusion and exclusion criteria were selected for the study. A male-to-female ratio of 1.2:1 within the age group 20-65 years (mean age 50.53 ± 9.98 years) having a duration of diabetes mellitus in the range of 0-16 years (mean duration 6.32 ± 3.61 years). Baseline characteristics are shown in Table 1.

**Table 1:** Baseline characteristics of variables included in the study LVDD- Left Ventricular Diastolic Dysfunction, HbA1c- Glycosylated Hemoglobin, FBS- Fasting Blood Sugar, PPBS- Post-Prandial Blood Sugar

VARIABLE	RANGE	LVDD PRESENT n (%) ABSENT n (%)		TOTAL n (100 %)
AGE	20 - 35	3 (30)	7 (70)	10
	36 - 50	18 (46.2)	21 (53.2)	39
	51 - 65	33 (64.7)	18 (35.3)	51
GENDER	MALE	28 (51.9)	26 (48.1)	54
	FEMALE	26 (56.5)	20 (43.5)	46
HbA1C	6.5 - 8 %	19 (43.2)	25 (56.8)	44
	8.1 - 10 %	5 (38.5)	8 (61.5)	13
	>10 %	30 (69.8)	13 (30.2)	43
FBS	<200 mg/dl	21 (63.6)	12 (36.4)	33
	200 - 300 mg/dl	30 (49.2)	31 (50.8)	61
	>300 mg/dl	3 (50)	3 (50)	6
PPBS	<300 mg/dl	9 (33.3)	18 (66.7)	27
	300 - 400 mg/dl	32 (56.1)	25 (43.9)	57
	401 – 500 mg/dl	13 (81.2)	3 (18.8)	16
LVDD	0 – 5 years	17 (38.6)	27 (61.4)	44
	5.1- 10 years	20 (57.1)	15 (42.9)	35
	>10 years	17 (81)	4 (19)	21

FBS, PPBS, and HbA1c were done. Echocardiography was done in all patients to evaluate left ventricular diastolic dysfunction. Out of 100 cases, 54 patients had LVDD and 46 patients had normal LVDD. LVDD was present in 54 (54%) of the cases and among them, 28 were males and 26 were females. There was no association found between the study groups and gender distribution shown. There is a varied difference in the prevalence of LVDD among different studies; this may be due to demographic factors and other risk factors. Most of the patients with normal left ventricular diastolic function were of age group 36-50 years (n=21, 38.89%) with a mean age of 48.02 ± 10.12 years. For patients in the LVDD group, most cases were of the age group interval 51-65 years (n=33, 61.11%) with a mean age of 52.67 ± 9.45 years. The mean HbA1c of the population with LVDD was seen as higher (11.07 ± 3.66%) as compared to the population with normal LVDD (9.11 ± 2.95%), which was found statistically significant (p-value=0.001). This denotes that a higher level of HbA1c in a patient with diabetes will have a higher incidence of LVDD. The mean FBS of the population with LVDD was found higher (231.52 ± 49.36) as compared to the population with normal LVDD (208.15 ± 42.01). The mean PPBS of the population with LVDD was also found higher (362.24 ± 62.68) as juxtaposed to patients with normal LVDD (313.54 ± 53.50) and the correlation was significant. This signifies that higher PPBS in patients with diabetes will have a higher incidence of LVDD.

Univariate logistic regression analysis is shown in Table 2. On applying univariate analysis, each unit of increase in age increased the risk of LVDD by 1.04 with 95% CI (1.002-1.09), P value 0.038. Similarly, every unit of increase in FBS increased the risk of LVDD by 1.01 with a 95% CI (1.002-1.021), P value of 0.014. Likewise, every unit of increase in PPBS increased the risk of LVDD by 1.01 with a 95% CI (1.005- 1.022), P value of 0.001. Similarly, every unit of increase of H bA1c increased the risk of LVDD by 21% (OR: 1.21 with 95% CI (1.05-1.38), P= 0.005). On the other hand, every year of increase in the duration of diabetes increased the risk of LVDD by 30% (OR, 1.30 with 95 % CI (1.14-1.48), P<0.001)).

**Table 2:** Univariate analysis to predict LVDD in patients’ diabetes, dependent variable: LVDD OR- Odds Ratio, CI- Confidence Interval, P value- Probability Value, FBS- Fasting Blood Sugar, PPBS- Post-Prandial Blood Sugar, HbA1c- Glycosylated Hemoglobin, LVDD- Left Ventricular Diastolic Dysfunction

	OR	Lower CI	Upper CI	P value
Gender	1.38	0.61	3.11	0.42
Age in years	1.04	1.002	1.09	0.038
FBS	1.01	1.002	1.021	0.014
PPBS	1.01	1.005	1.022	0.001
HbA1C	1.21	1.05	1.38	0.005
Duration in years	1.30	1.14	1.48	<0.001

To determine the independent effect of the variable included in the study, multivariate analysis after removing FBS and PPBS due to collinear effects was done (Table 3).

**Table 3:** Multivariable analysis to determine the independent effect of variables for LVDD OR- Odds Ratio, CI- Confidence Interval, P Value- Probability Value, HbA1C- Glycosylated Hemoglobin

	OR	Lower CI	Upper CI	P value
Gender	1.34	0.50	3.56	0.54
Age in years	0.95	0.88	1.02	0.20
HbA1C	1.26	1.08	1.48	0.003
Duration in years	1.48	1.20	1.82	<0.001

**Figure 1:**
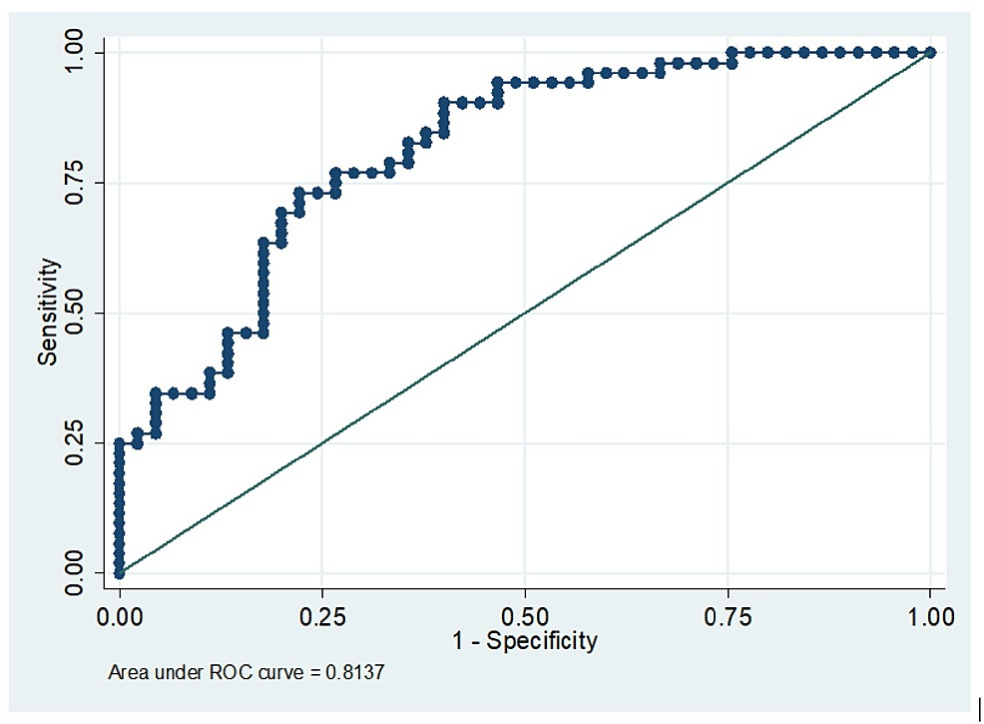
Logistic ROC analysis for a multivariable model ROC- Receiver Operating Characteristic Curve

We observed that HbA1C was found to be a significant variable for the 26% higher risk for LVDD (OR 1.26 with CI 95% (1.08-1.48), P= 0.003). The duration was also found to be a significant variable for increasing risk for LVDD (OR 1.48 with CI 95 % (1.20-1.82) P value <0.001). The predictive accuracy for the model was also clinically acceptable measured by area under the curve = 81% (Figure 1).

## Discussion

The higher the age groups of the study population, the more the incidence of LVDD [25]. The majority of patients with normal LVDD were in the age group 36-50 years (n=21, 38.89%) with a mean age of 48.02 ± 10.12 years. In patients in the LVDD group, most of them were in the age group 51-65 years (n=33, 61.11%) with a mean age of 52.67 ± 9.45 years. In one study that was followed for four years, the prevalence of diastolic dysfunction increased with an increase in age, and diastolic dysfunction was associated with the development of heart failure during six years of subsequent follow-up [26]. There was no association found between the study groups and gender distribution.

Among 45 (80%) patients out of 55 LVDD patients, 15 (27%) patients had Grade 1 diastolic dysfunction, 26 (47%) had Grade 2 diastolic dysfunction, and four (7%) patients had Grade 3 diastolic dysfunction. In this study, a comparison was further made between the population with LVDD and the population without LVDD using various parameters like FBS, PPBS, and HbA1c levels. Mean HbA1c among the population with LVDD was found higher (11.07 ± 3.66%) as compared to the population with normal LVDD (9.11 ± 2.95%), which was statistically found significant (p-value=0.004). This signifies that the higher the HbA1c of a patient with diabetes, the higher the incidence of LVDD. Similarly, a study of 100 cases of newly diagnosed diabetes mellitus found that the mean HbA1c of subjects with LVDD was 7.95 ±1.09 and of subjects without LVDD was 7.21 ± 1.22. This study also concluded that the HbA1c level is positively associated with the incidence of LVDD in the diabetic population as the mean HbA1c of the population with LVDD was higher than the population with normal LVDD [27]. An article published earlier found that among diabetic cases, 9.09% of cases with an HbA1c range of 6-7%, 33.33% of cases with an HbA1c range of 7.1-8%, 100% of cases with an HbA1c range >8.1% were showing diastolic dysfunction that was statistically significant [28]. The mean FBS of the population with LVDD was found to be high (231.52 ± 49.36) as compared to the population with normal LVDD (208.15 ± 42.01), which was statistically found significant (p-value=0.013). The mean PPBS of the population with LVDD was also found high (362.24 ± 62.68) as juxtaposed to the population without LVDD (313.54 ± 53.50). The correlation was found significant using an unpaired t-test (p-value=0.001). This denotes that higher FBS and PPBS in patients with diabetes will have a higher incidence of LVDD. A study published earlier included 101 asymptomatic patients of type 2 diabetes without overt heart disease; the LVDD was significantly and positively correlated with the increased fasting blood sugar concentration [29].

**Figure 2:**
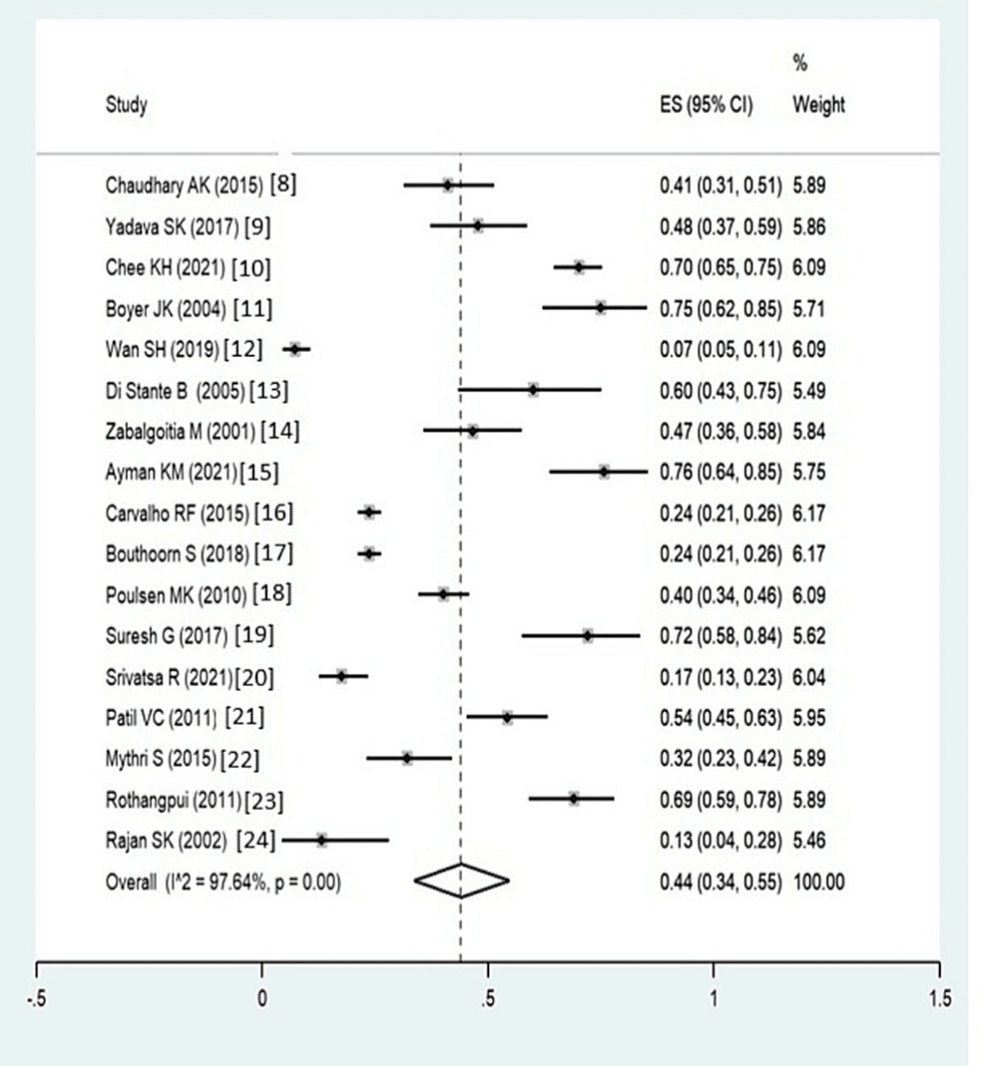
Forest plot Forest plot of the pooled proportion of left ventricular dysfunction in diabetes patients [8-24]

A forest plot drawn for LVDD from different studies shows the cumulative prevalence of LVDD to be 44% (95% CI: 34% to 55%) (Figure 2). However, there are certain limitations of this study, including a lack of comparative echocardiographic findings across the LVDD and non-LVDD groups and a higher mean age of the study population; these should be taken care of in future studies.

## Conclusions

Patients who have higher glycosylated hemoglobin levels are linked to having a higher risk of left ventricular diastolic dysfunction in patients with type 2 diabetes. The combination of diabetes and left ventricular dysfunction can act as a double-edged sword. This leads to increased morbidity and mortality in those patients in whom it is not identified timely and appropriate measures are not taken. Our study emphasizes the need to screen intermittently asymptomatic diabetic patients for diastolic dysfunction through a Doppler echocardiography so that timely action can be taken.

Hence, we conclude that in the future, left ventricular diastolic dysfunction can be detected at the earliest by screening raised HbA1C in type 2 diabetes. Thereby, necessary action can be taken at an earlier stage to prevent mortality and morbidity.
